# Competitive outcome of multiple infections in a behavior‐manipulating virus/wasp interaction

**DOI:** 10.1002/ece3.1749

**Published:** 2015-12-05

**Authors:** Julien Martinez, Frédéric Fleury, Julien Varaldi

**Affiliations:** ^1^ Department of GeneticsUniversity of CambridgeCambridgeCB2 3EHUK; ^2^ Laboratoire de Biométrie et Biologie EvolutiveUniversité de Lyon69000LyonFrance; ^3^ Centre National de la Recherche ScientifiqueUnité Mixte de Recherche 5558Université Lyon 169622 VilleurbanneFrance

**Keywords:** coinfection, *Drosophila* parasitoid, *Leptopilina boulardi* Filamentous Virus, within‐host competition

## Abstract

Infections by multiple parasites are common in nature and may impact the evolution of host–parasite interactions. We investigated the existence of multiple infections involving the DNA virus LbFV and the *Drosophila* parasitoid *Leptopilina boulardi*. This vertically transmitted virus forces infected females to lay their eggs in already parasitized *Drosophila* larvae (a behavior called superparasitism), thus favoring its spread through horizontal transmission. Previous theoretical work indicated that the evolution of the level of the manipulation strongly depends on whether infected parasitoids can be re‐infected or not. Here, we describe a strain of LbFV that differs from the reference strain by showing a deletion within the locus used for PCR detection. We used this polymorphism to test for the existence of multiple infections in this system. Viral strains did not differ on their vertical or horizontal transmission rates nor on the way they affect the parasitoid's phenotype, including their ability to manipulate behavior. Although already infected parasitoids were much less susceptible to new infection than uninfected ones, frequent coinfection was detected. However, following coinfection, competition between viral strains led to the rapid elimination of one strain or the other after a few generations of vertical transmission. We discuss the implications of these results for the evolution of the behavioral manipulation.

## Introduction

The evolution of parasite virulence has been extensively studied using various theoretical models integrating different levels of complexity (Alizon et al. 2009). A major hypothesis of these models is that parasites face a trade‐off between their virulence and their transmission rate and should evolve toward levels of virulence that maximize their spread in a population of hosts (Anderson and May 1982; Ewald 1987; Van Baalen and Sabellis 1995; Frank 1996; Alizon and van Baalen 2005). One prediction of the trade‐off hypothesis is that more opportunities for horizontal transmission (between hosts) should select for higher virulence. However, increasing opportunities for horizontal transmission also raises the equilibrium prevalence of the parasite (Lipsitch et al. 1995). In cases where infected hosts cannot be reinfected, such an increase in the parasite prevalence creates an epidemiological feedback that selects for lower virulence (Lipsitch et al. 1996). This feedback becomes even stronger when the parasite can also persist in host lineages through efficient vertical transmission (from mother to offspring). In that case, at high equilibrium prevalence, the fitness of the parasite becomes mostly dependent on its vertical transmission efficiency.

However, such predictions do not take into account the possibility for an individual host to be infected with multiple parasite strains. Multiple infections are common in host–parasite associations (López‐Ferber et al. 2003; Mouton et al. 2003; Ben‐Ami et al. 2008; Salvaudon et al. 2013) and are expected to have major consequences on the evolution of parasite virulence (Nowak and May 1994; May and Nowak 1995; Van Baalen and Sabellis 1995; Frank 1996; Alizon 2013). On the one hand, the fact that infected hosts remain susceptible to new infections should diminish the epidemiological feedback of parasite prevalence on the evolution of virulence. On the other hand, multiple infections allow interaction between parasites within the host. Because a host is a limited resource, competition between parasite strains or species within the host leads to the classical “tragedy of the commons” problem that favors faster replication rates and thus greater virulence (Hardin 1968; Frank 1996). Conversely, it has also been suggested that coinfection may favor the evolution of lower virulence when virulence originates from the production of public goods by the parasites (Turner et al. 1999; Brown et al. 2002). For instance, parasites may secrete compounds that modify their host niche in a way that benefit their own fitness through the expression of a behavioral manipulation (Biron and Loxdale 2013). In the context of multiple infections, parasite strains producing the extended phenotype can be exploited by nonproducing cheater strains which benefit from the collective action of the group without paying the associated cost (Chao et al. 2000; Brown 2001; Smith 2001; Brown et al. 2002; Buckling and Brockhurst 2008). Under this scenario, a reduced investment in the production of the extended phenotype is expected in case of multiple infections (Brown 1999; Vickery and Poulin 2010).

Here, we investigated the existence of multiple infections in the maternally transmitted DNA virus LbFV that specifically infects and manipulates the behavior of the Hymenopteran parasitoid *Leptopilina boulardi* (Varaldi et al. 2003, 2006c). The parasitoid attacks *Drosophila* larvae by laying its eggs inside their body. The parasitoid larva consumes all the tissues of the *Drosophila* larva, leading to its death and to the emergence of a parasitoid adult. Virus‐free parasitoid females lay a single egg into each encountered *Drosophila* larva but usually reject already parasitized ones. In contrast, LbFV‐infected females readily superparasitize, that is, lay eggs into already parasitized larvae, exposing their offspring to a strong competition as only one parasitoid can emerge from a single *Drosophila* larva. Note that we will use “infected” to refer to parasitoids harboring the virus and “parasitized” to refer to *Drosophila* larvae harboring parasitoid(s). Interestingly, superparasitism allows the virus to be horizontally transmitted between parasitoid offspring sharing the same *Drosophila* larva. By a theoretical approach, Gandon et al. (2006) derived the evolutionary stable strategy (ESS) of superparasitism (rate of acceptance of parasitized larvae) both from the parasitoid (in the absence of virus) and from the virus point of view. The model indicates that the ESS is always higher for the virus compared to the parasitoid (who may still benefit from superparasitism under harsh competition for hosts), suggesting that the virus‐induced behavior is a case of behavioral manipulation. This initial model also predicted that the optimal level of manipulation is strongly dependent on the virus prevalence which has been found to vary to a great extent in natural populations (Patot et al. 2010). Basically, when infected parasitoids cannot be re‐infected, the virus should encounter fewer susceptible hosts as its prevalence increases, which in turn selects for lower level of manipulation due to the epidemiological feedback. However, an extension of this model including superinfection (replacement of a strain by another one through within‐host competition) showed that within‐host competition is a major factor affecting the evolution of behavior manipulation in this system (Varaldi et al. 2009). In this case, because infected parasitoids still represent potential hosts for the virus (contrary to the previous case without superinfection), selection favors an increased investment in behavioral manipulation (Varaldi et al. 2009).

In this study, we describe a natural LbFV strain showing a short deletion (111 bp) in the gene classically used for PCR detection. We used this sequence polymorphism to test whether and how an epidemiological feedback may be at play in this virus‐wasp system. To address this, we first compared the reference and the deleted viral strains by measuring their respective rates of transmission (vertical and horizontal) and their effects on parasitoid life‐history traits including the intensity of the manipulation. We also tested whether one viral strain can replace a resident one (superinfection) and whether several viral strains can coexist within the same parasitoid (coinfection), together with the fate of coinfection along generations. We discuss our results in the light of theoretical predictions on the evolution of the behavioral manipulation.

## Material and Methods

### PCR detection of LbFV

We detected the presence of *Leptopilina boulardi* Filamentous Virus (LbFV) by amplifying a genomic sequence initially identified by comparing the transcriptome of infected and uninfected parasitoid lines using suppressive subtractive hybridization (accession number: FM876312, Patot et al. 2009). The analysis of the mRNA revealed a potential open reading frame encoding a 205 amino acid protein. Using the protocol described in Patot et al. 2009, we detected the presence of LbFV with primers 102‐F and 500‐R in a multiplex PCR with insect primers RPS2‐F and RPS2‐R amplifying a parasitoid ribosomal protein gene to control for the quality of DNA extractions.

### Viral strains and parasitoid genotype

The PCR setup described above typically leads to the amplification of a 399‐bp viral genome fragment in infected parasitoids (primers 102‐F and 500‐R, see above). In September 2007, a new strain of LbFV was discovered after the sampling of *L. boulardi* in a French orchard (Avignon). Among the 21 parasitoid lines collected from the same trap, 16 displayed a positive PCR amplification. Among these infected lines, one of them displayed a unique and unusual amplification of a 288‐bp fragment. Moreover, nine other lines showed this shorter fragment along with the typical 399‐bp fragment suggesting that parasitoids can be coinfected by several strains at a time. The unusual PCR product was sequenced using the Sanger method and revealed a 111‐bp deletion in the LbFV locus (accession number: JX455824). The reference viral strain LbFV1 (399 bp), as well as the deleted viral strain LbFV2 (288 bp), was maintained in the laboratory on the inbred parasitoid line Av3 (originating from Avignon, 5 generations of sib‐mating, Martinez et al. 2012b). All parasitoids were reared under a 12L:12D photoperiod at 26°C using a laboratory line of *Drosophila melanogaster* originating from Ste‐Foy‐lès‐Lyon (France). *Drosophila* larvae were fed with a standard diet (David 1962).

### Experimental setup for horizontal transmission

Horizontal transmission of LbFV occurs exclusively in the context of superparasitism (Varaldi et al. 2003, 2006b). As *L. boulardi* is a haplo‐diploid species, fertilized eggs develop into females whereas unfertilized ones give rise to males. Hence, virus horizontal transmission can easily be monitored by obtaining *Drosophila* hosts successively parasitized by a mated mother (recipient line) and a virgin infected mother (donor line). As females emerging from these hosts are necessarily offspring of the mated mother, the occurrence of horizontal transmission is simply tested by PCR detection of the virus in those females. In practice, one mated parasitoid female (1‐ to 2‐day‐old) of the recipient line of interest was placed with 100 *Drosophila* eggs for 24 h in a rearing vial. Then, the female was removed and replaced by four virgin females (2‐ to 3‐day‐old) of the donor line of interest for an additional 24 h. The success of horizontal transmission was then evaluated using the PCR test on emerging females. For each vial, mothers of the recipient and donor lines were PCR checked for viral infection.

In a first experiment, we quantified and compared the ability of both viral strains (LbFV1 and LbFV2) to be horizontally transmitted on a single genetic background of *L. boulardi* (Av3) that was either uninfected or already infected with one of the viral strains (Table 1). Seven independent replicates (= vials) were performed in each combination. From this first experiment, we obtained two coinfected parasitoid lines, one obtained with the transfer from Av3[LbFV1] to Av3[LbFV2] and the other with the transfer from Av3[LbFV2] to Av3[LbFV1]. Because no statistical differences were found between these two coinfected lines in the subsequent experiments for any tested traits, the data obtained were pooled and analyzed as a unique coinfected line Av3[LbFV1+LbFV2].

**Table 1:** Different combinations of LbFV horizontal transfer

Experiment	Donor line^a^		Recipient line^a^	Number of replicates	Total number of female offspring tested
1	Av3[LbFV1]	→	Av3	*n* = 7	*n* = 24
1	Av3[LbFV2]	→	Av3	*n* = 7	*n* = 23
1	Av3[LbFV1]	→	Av3[LbFV2]	*n* = 7	*n* = 33
1	Av3[LbFV2]	→	Av3[LbFV1]	*n* = 7	*n* = 27
2	Av3[LbFV1]	→	Av3	*n* = 4	*n* = 18
2	Av3[LbFV1+LbFV2]	→	Av3	*n* = 4	*n* = 13

a Av3: parasitoid nuclear background. Viral infection status in brackets.

In a second experiment, we tested whether the two viral strains can be co‐transmitted horizontally using the coinfected line Av3[LbFV1+LbFV2] as the donor line and the uninfected line Av3 as the recipient line (Table 1). The transfer from Av3[LbFV1] to uninfected parasitoids in this second experiment was used as a control for horizontal transmission efficiency.

Successful horizontal transmission depends on the actual rate of transmission (from one parasitoid egg to another in the situation of superparasitism) but also on the opportunities for horizontal transmission (the frequency and the intensity of superparasitism). Therefore, superparasitism intensity was quantified by dissecting five *Drosophila* pupae in each replicate. Parasitoid eggs were counted under a stereomicroscope, and for a given replicate, superparasitism intensity was defined as the mean number of parasitoid eggs per parasitized *Drosophila* larva. Parasitism rate, that is, the proportion of parasitized larvae, was also measured for each vial, as explained below.

### Vertical transmission and segregation of the coinfection

Two generations after the experiment 1 (horizontal transmission experiment), lines Av3[LbFV1], Av3[LbFV2], and Av3[LbFV1+LbFV2] were used to measure the vertical transmission rate of each viral strain from one generation to the next. One‐ to two‐day‐old infected females (PCR verification; *n* = 10, 10 and 20 for Av3[LbFV1], Av3[LbFV2], and Av3[LbFV1+LbFV2], respectively) were individually placed for 24 h in vials containing *Drosophila* larvae hatched from 100 eggs. In such conditions, superparasitism frequency is low enough to render horizontal transmission negligible. PCR detection of LbFV strains was performed on female offspring emerging from these vials (*n* = 46 to 100 per condition).

We further tested the within‐host competitive ability of LbFV1 and LbFV2 over four generations of vertical transmission. To achieve this, we created 40 coinfected isofemale lines originating from the line Av3[LbFV1+LbFV2] (PCR checked at generation 0). A single female per isofemale line was kept to establish the next generation. The infection status of the lines was then checked by PCR detection on adult females of the fourth generation. The proportion of false‐negative among PCR tests was evaluated by testing viral infection in eleven lines of the generation 2 that were negative for infection at the previous generation, either for one viral strain or for both. All eleven cases of apparent infection loss at the generation 1 (no LbFV1 = 2, no LbFV2 = 3, uninfected = 6) were confirmed at the next generation, suggesting that the proportion of false‐negative among PCR tests is low.

### Modelization of vertical transmission in coinfection under the hypothesis of independent transmission

Based on the estimates of vertical transmission of LbFV1 and LbFV2 in single infection obtained in the previous experiment, we constructed a simple Markovian model for the vertical transmission of strains in the context of coinfection, under the hypothesis that the transmission of each strain is independent of the presence of the other (i.e., absence of within‐host competition for vertical transmission). Under this hypothesis, if we call P1 the vertical transmission efficiency of LbFV1 in single infection and P2 the vertical transmission efficiency of LbFV2, the probabilities of state transitions can be expressed in a transition matrix as follows:

$$M=\left[\begin{array}{cccc} P_{\mathrm{uninfected}>\mathrm{uninfected}} & P_{\mathrm{uninfected}>\mathrm{LbFV}1} & P_{\mathrm{uninfected}>\mathrm{LbFV}2} & P_{\mathrm{uninfected}>\mathrm{coinfected}}\\ P_{\mathrm{LbFV}1>\mathrm{uninfected}} & P_{\mathrm{LbFV}1>\mathrm{LbFV}1} & P_{\mathrm{LbFV}1>\mathrm{LbFV}2} & P_{\mathrm{LbFV}1>\mathrm{coinfected}}\\ P_{\mathrm{LbFV}2>\mathrm{uninfected}} & P_{\mathrm{LbFV}2>\mathrm{LbFV}1} & P_{\mathrm{LbFV}2>\mathrm{LbFV}2} & P_{\mathrm{LbFV}2>\mathrm{coinfected}}\\ P_{\mathrm{coinfected}>\mathrm{uninfected}} & P_{\mathrm{coinfected}>\mathrm{LbFV}1} & P_{\mathrm{coinfected}>\mathrm{LbFV}2} & P_{\mathrm{coinfected}>\mathrm{coinfected}} \end{array}\right]$$

$$M=\left[\begin{array}{cccc} 1 & 0 & 0 & 0\\ 1-P_{1} & P_{1} & 0 & 0\\ 1-P_{2} & 0 & P_{2} & 0\\ \left(1-P_{1}\right)\times \left(1-P_{2}\right) & P_{1}\times \left(1-P_{2}\right) & \left(1-P_{1}\right)\times P_{2} & P_{1}\times P_{2} \end{array}\right]$$

with *P*_infection status *x > *infection status *y*_ being the probability that a mother with infection status *x* produces offspring with infection status *y*. Any distribution of infection statuses at a given generation can be expressed as:

$$G_{n}=\left[\begin{array}{cccc} P_{\mathrm{uninfected}} & P_{\mathrm{LbFV}1} & P_{\mathrm{LbFV}2} & P_{\mathrm{coinfected}} \end{array}\right]$$

and the expected distribution at generation *n* + 1 may simply be obtained this way:

$$G_{n+1}=G_{n}\cdot M$$

We ran the Markov chain independently forty times for four generations to mimic the conditions of the previous experiment (40 lines, four generations). At each generation, transmission events were randomly drawn from a binomial distribution with the *ad hoc* probability of success. Then, 1000 such simulations were run to generate 1000 expected distributions after four generations of vertical transmission. The observed number of coinfected lines (out of the 40) was then placed within the distribution of the expected numbers and a *P*‐value was calculated (See supporting information for the R script).

### Effect of LbFV strains on parasitoid life‐history traits

As the fitness of a particular viral strain depends on its transmission rate but also on its effect on the parasitoid's phenotype, we measured several parasitoid life‐history traits for the different infection statuses. Previous works showed that LbFV increases the tendency of adult parasitoid females to accept superparasitism without detectable effect on other behavioral traits or on female survival (Varaldi et al. 2003, 2006b, 2009). In addition, it has a slight positive effect on egg load (≈ + 10%) and a weak negative effect on tibia length, locomotor activity and development time (Varaldi et al. 2005).

Superparasitism behavior was measured as described in Varaldi et al. 2003. One‐ to two‐day‐old parasitoid females (*n* = 17, 13, 18, and 40 for A3, A3[LbFV1], A3[LbFV2], and A3[LbFV1+LbFV2], respectively) were individually placed with ten *D. melanogaster* 1st instar larvae in a 5‐cm diameter Petri dish consisting of a thin yeast spot poured on an agar layer. After one night at 26°C, females were removed and individually frozen for later PCR detection of LbFV. Three *Drosophila* larvae per parasitoid female were dissected under a stereomicroscope in order to count parasitoid eggs. Superparasitism intensity for a given female was defined as the mean number of parasitoid eggs laid per parasitized *Drosophila* larva.

In addition, for each of the four infection statuses, we measured the overall fitness of females by putting one‐ to two‐day‐old females (*n* = 14, 10, 16, and 24 for A3, A3[LbFV1], A3[LbFV2], and A3[LbFV1+LbFV2], respectively) for 24 h in a rearing vial containing *Drosophila* larvae hatched from 100 eggs deposited the day before. Parasitoid females were then frozen for later PCR detection of LbFV. Additionally, ten control vials without parasitoids were prepared to measure the natural mortality of *Drosophila* hosts. All emerged flies and parasitoid offspring were collected and counted daily. The parasitism rate for a given female (proportion of parasitized *Drosophila* larvae) was estimated by comparing the number of adult flies emerging from parasitized vials to the mean number of flies emerging from the parasitoid‐free control vials, as described in Martinez et al. 2012a;. We estimated the parasitoid sex ratio as the proportion of males in the progeny, as well as the mean development time of parasitoid females and the developmental parasitoid success. This latter parameter is defined as the proportion of parasitized *Drosophila* larvae that give rise to adult parasitoids and thus depends on the parasitism rate estimate (Martinez et al. 2012a). Finally, the egg load and tibia length of five‐day‐old parasitoid females fed with honey were also measured according to the protocol described in Martinez et al. 2012b (*n* = 8, 8, 8, and 16 for A3, A3[LbFV1], A3[LbFV2], and A3[LbFV1+LbFV2], respectively).

### Statistical analysis

**Figure 1:**
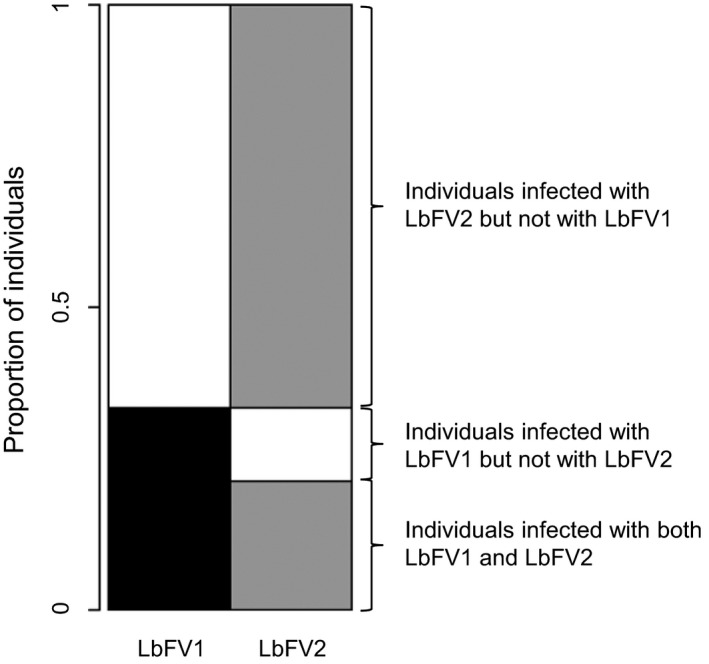
Example of data‐visualization of multiple infections using the R package Mondrian. Individuals are represented by horizontal lines, and each column represents one viral strain. Horizontal lines (representing individuals) are reordered to optimize the reading. LbFV1 is always represented in black, LbFV2 in gray, and uninfected individuals in white.

All statistical analyses were carried out using R (R Core Team, 2013). Proportions (of uninfected, infected by LbFV1, LbFV2 or both) were analyzed using generalized linear models with a binomial error. In these models, the viral strain and the infection treatment were treated as fixed effects. When necessary, we included a random effect taking into account the possible effect of the replicate vial using the R package lme4. In the superparasitism experiment, the egg distribution among host larvae was analyzed using a Kolmogorov–Smirnov nonparametric test. Other phenotypic traits were analyzed using linear models with appropriate transformation if needed, in order to reach the assumptions of normality and homoscedasticity. Simulations based on the Markov chain model were used to infer *P*‐values by counting the number of simulations giving more extreme simulations compared to the observed value divided by the total number of simulations (two‐sided test). Finally, infection data are represented using the R package called “Mondrian” in reference to the famous painter (Siberchicot, unpublished data, see Fig. 1 for an example).

## Results

### Characterization of LbFV strains

**Figure 2:**
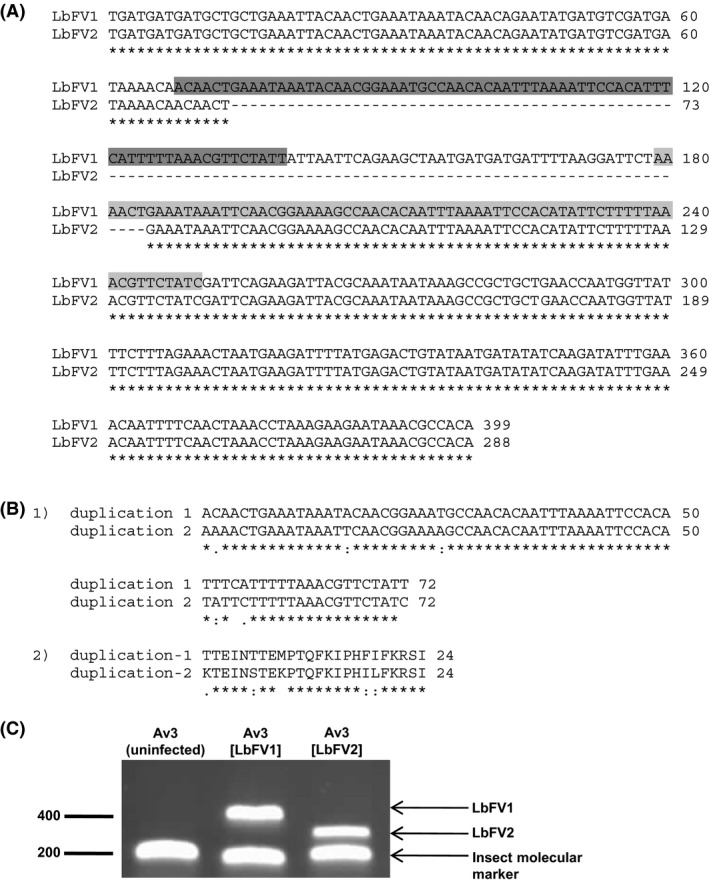
(A) Alignment of LbFV1 and LbFV2 molecular markers amplified with primers 500‐R and 102‐F (Accession Numbers: FM876312 and JX455824, respectively). Stars indicate homology. Dark and light gray bars indicate two regions showing high sequence similarity between them, suggesting a putative duplication. (B) Nucleotide (1) and protein (2) alignments of the putative duplications. (C) Agarose gel electrophoresis of PCR products obtained on individual parasitoids.

The sequencing of the viral molecular marker revealed the presence of two viral strains in *L. boulardi*, LbFV1 described in a previous study (Patot et al. 2009) and the new strain LbFV2 that shows a 111 bp deletion on the molecular marker (Fig. 2A and C). The deleted sequence is located in a region of putative duplication with two consecutive repetitions of a 72‐bp motif (Fig. 2A and B). In the reference strain LbFV1, these DNA repetitions are separated by a 39‐bp sequence which is a multiple of 3. Consequently, the predicted amino acid sequence also includes amino acid repetitions (Fig. 2B) and the deletion does not disrupt the predicted open reading frame. Therefore, LbFV2 sequence is expected to encode a 168 amino acid protein, whereas LbFV1 sequence encodes a 205 amino acid protein.

**Figure 3:**
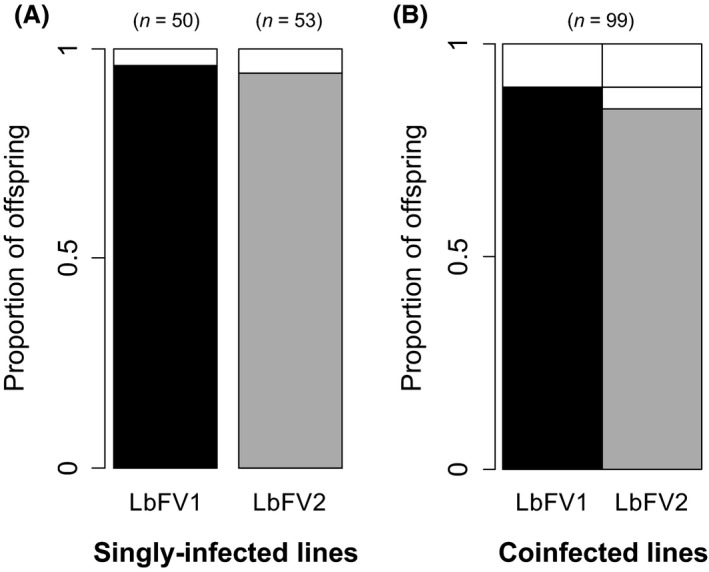
Frequency distribution of infection statuses among progenies of (A) singly infected females and (B) coinfected females. Black, gray, and white bars stand for LbFV1‐, LbFV2‐infected and uninfected offspring, respectively. The number of offspring tested is shown at the top of each graph. The proportion of a given viral strain is obtained by summing vertically the corresponding color. For coinfected lines, horizontal reading gives the proportion of their offspring sharing a given infection status.

The vertical transmission rate of both viral strains was quantified in the context of single infection. Both strains showed very high transmission from mother to offspring with no significant differences between them (96% for LbFV1 and 94.3% for LbFV2, generalized linear mixed‐effect model; Dev = 0.03; *P *=* *0.86; Fig. 3A).

**Figure 4:**
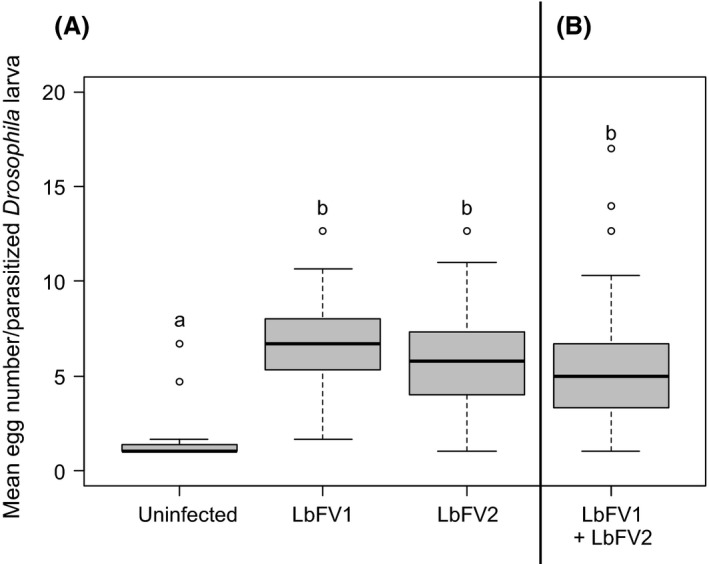
Superparasitism intensity of the different infection statuses. (A) uninfected versus singly infected parasitoid females. (B) coinfected females. Different letters indicate significant differences (pairwise *t*‐test on log‐transformed data with Bonferroni correction for multiple comparisons, *α *= 0.008).

We also measured the phenotypic effect of both viral strains on superparasitism. As expected, the superparasitism behavior depended on the infection status of females (log‐transformed data; *F*_3,83 _= 18.7; *P *<* *0.0001; Fig. 4A): infected females superparasitized much more than uninfected ones (pairwise *t*‐test with Bonferroni correction for multiple comparisons, Fig. 4A). There was, however, no difference in the superparasitism intensity induced by LbFV1 and LbFV2 (Fig. 4A). There was also no difference in the distribution of parasitoid eggs among host larvae between the two viral strains (Kolmogorov–Smirnov test; *P *=* *0.49).

### Coinfection arises through horizontal transmission

**Figure 5:**
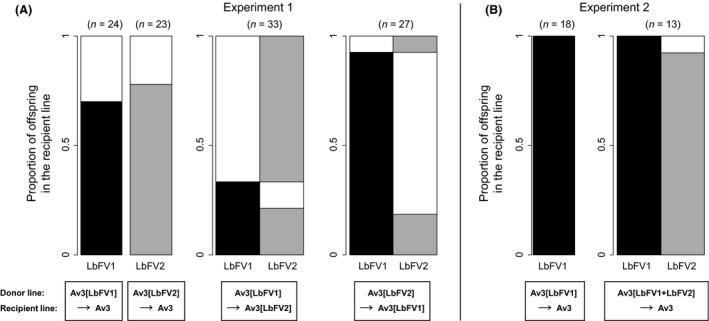
Distribution of infection statuses in horizontal transmission experiments. (A) experiment 1; horizontal transmission from singly infected parasitoid females. (B) experiment 2: horizontal transmission from singly infected and coinfected parasitoid females. Different colors stand for different infection statuses in emerged offspring of the recipient line: uninfected (white), infected with LbFV1 (black), or infected with LbFV2 (gray). The number of offspring tested is shown at the top of each graph. The proportion of a given viral strain is obtained by summing vertically the corresponding color. In case of infected recipient line (A) or when the donor line is coinfected (B), horizontal reading gives the proportion of offspring in the recipient line sharing a given infection status.

We tested the horizontal transmission of both viral strains on uninfected and infected recipient wasps. First, both LbFV strains were massively horizontally transmitted to the uninfected parasitoid recipient line (Fig. 5A). Importantly, both viral strains were horizontally transferred with similar rates independently of the infection status of the recipient wasp (uninfected recipient: 71% for LbFV1 and 78% for LbFV2; already infected recipient: 33% for LbFV1 and 26% for LbFV2; Table 2). However, the successful horizontal transfer of both strains strongly depended on whether the recipient line was already infected or not (Table 2). Indeed, the efficiency of successful horizontal transmission into already infected parasitoid lines was about twofold lower than into the uninfected recipient line (Fig. 5A).

**Table 2:** Generalized linear mixed‐effect model on horizontal transmission rates in experiment 1

Variable	Deviance	df	*P*
Viral strain	0.039	1	0.84
Treatment (uninfected vs. infected recipient line)	14.25	1	0.0002
Viral strain‐by‐treatment interaction	0.85	1	0.36

Although the presence of the virus limited the infection with the other viral strain, horizontal transmission to already infected wasps did occur in a significant number of cases. In these cases, 64% and 71% of the parasitoids were infected with both the resident and the “invasive” strains, respectively, for the transfer of LbFV1 and LbFV2. These proportions of coinfected individuals were not significantly different between the two viral strains (generalized linear mixed‐effect model; deviance = 0.07; *P *=* *0.79). In very few cases, we only detected the horizontally transmitted strain and not the resident one (4/33 for the transfer Av3[LbFV1]>Av3[LbFV2]; 2/27 for the transfer Av3[LbFV2]>Av3[LbFV1]) suggesting replacement of the resident strain through within‐host competition (superinfection). However, note that these apparent rare superinfection events may also be due to a concomitant loss of the resident strain by incomplete vertical transmission.

Because horizontal transmission efficiency depends not only on the rate of transmission *per se* but also on the frequency of horizontal transmission opportunities, we measured proxies for horizontal transmission opportunities during this experiment: the parasitism rate and the superparasitism intensity. There was no difference among treatments on both the parasitism rate (arcsine‐transformed data, *F*_3,24 _= 1.17; *P *=* *0.34) nor on the superparasitism intensity (log‐transformed data, *F*_3,24_ = 1.9; *P *=* *0.16), suggesting that the differences in horizontal transmission efficiency that we reported above were due to differences in the rate of transmission *per se* rather than differences in horizontal transmission opportunities. In other words, this means that superparasitism events (that are necessary for horizontal transmission) were as frequent in all treatments, even though horizontal transmission was reduced by twofold when the recipient line was already infected.

In another experiment (experiment 2), we tested whether viral strains coinfecting the same parasitoid could be jointly horizontally transmitted to uninfected parasitoids. We found that all tested offspring of the recipient line were infected with LbFV1 and 92% of them (12 among 13 tested females) were coinfected. The horizontal transmission of the coinfection was not significantly different from the transmission of LbFV1 in the case of single infection (generalized linear mixed‐effect model; *deviance *= 1.78; *P *=* *0.18; Fig. 5B). As we previously shown that LbFV1 and LbFV2 do not differ on their horizontal transmission success in case of single infection, this suggests the absence of competition between viral strains for horizontal transmission.

### Viral strains compete for vertical transmission

We used the coinfected lines obtained from the previous experiment to investigate the outcome of the competition between viral strains across wasp generations. After one generation of vertical transmission, LbFV1 and LbFV2 were transmitted to 89.8% and 84.8% of the offspring, respectively (including co‐transmission). These proportions were not significantly different (Generalized linear model; *z *=* *−1.06; *P *=* *0.29) indicating that there is no competitive asymmetry between the strains (Fig. 3B). To test the hypothesis of competition for vertical transmission, we calculated the expected proportion of co‐transmission of LbFV1 and LbFV2, under the hypothesis of independent transmission. Under this hypothesis, the expected proportion of co‐transmission is then simply the product of single infection transmission rates (estimated in Fig. 3A), that is, 96% × 94.3% = 90.5%. The observed proportion of coinfection was slightly lower than this expected value, but this difference was only marginally significant (84.8%; exact binomial test: *P *=* *0.06).

**Figure 6:**
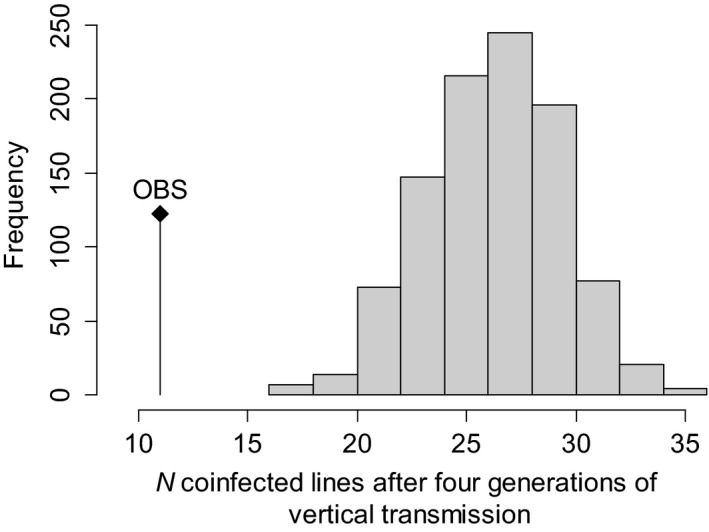
Expected distribution of coinfection after four generations of vertical transmission under the hypothesis of independent transmission of viral strains. The expected number of coinfected lines was inferred from a Markovian model (see methods). OBS, observed number of coinfected lines.

We further tested the stability of the coinfection and the fitness of the viral strains through four generations of vertical transmission, starting from the coinfected line Av3[LbFV1+LbFV2]. Among the 40 coinfected lines initially created, 85% were still infected by at least one viral strain after four generations of vertical transmission. Importantly, there was no evidence of competitive asymmetry in vertical transmission rates among viral strains as LbFV1 and LbFV2 infected a similar proportion of the lines, respectively, 55% and 57.5% (generalized linear model; *z *=* *0.22; *P *=* *0.82). Among the infected lines, 27.5% were still coinfected after four generations of vertical transmission. To test the hypothesis of competition for vertical transmission among viral strains, we compared the observed number of coinfected lines after four generations to the expected number of coinfected lines at the same generation based on the hypothesis of independent transmission of each viral strain. The expected number of coinfected lines was deduced from a simple Markov chain model that was run 1000 times. The distribution of the expected number of coinfected lines and the observed value are shown in Figure 6. The observed number of coinfected lines was significantly lower (*P *<* *0.001) than the expected number (see Supporting information for details) supporting the hypothesis that viral strains compete for vertical transmission.

### Coinfection does not affect the expression of the extended phenotype

We tested whether the intensity of the manipulation could be affected by the coinfection. As expected, the coinfected lines superparasitized more than the uninfected control (Fig. 4A and B). However, there was no difference between the coinfected line and the two singly infected lines (Fig. 4A and B). Moreover, the distributions of parasitoid eggs among host larvae were not significantly different between singly infected and coinfected parasitoid females (Kolmogorov–Smirnov test; A3[LbFV1] versus A3[LbFV1+LbFV2]: *P *=* *0.21; A3[LbFV2] versus A3[LbFV1+LbFV2]: *P *=* *0.62).

Other parasitoid life‐history traits were also measured in order to test for potential cost induced by single infection and coinfection. No difference could be attributed to the infection status of the parasitoids for any tested traits (Table 3).

**Table 3:** Analysis of the effect of the infection status on different parasitoid life‐history traits

Life‐history trait	Source of variation	*F* ratio	*P*
Parasitism rate (arcsine‐transformed data)	Infection status	*F*_3,54 _= 2.71	0.05
Parasitoid developmental success	Infection status	*F*_3,54 _= 1.34	0.27
Sex ratio	Infection status	*F*_3,54_ = 1.27	0.29
Mean development time	Infection status	*F*_3,54_ = 2.12	0.11
Egg load	Infection status	*F*_3,31 _= 2.09	0.12
	Tibia length	*F*_*1*,31 _= 41.88	<0.0001
	Interaction	*F*_*3*,31 _= 0.61	0.61
Tibia length	Infection status	*F*_3,31 _= 0.76	0.52

## Discussion

Understanding the epidemiology of a parasite is a prerequisite to make predictions on the evolution of its virulence. Particularly, the occurrence and the competitive outcome of multiple infections can deeply shape the effect of a parasite on its host (Nowak and May 1994; Van Baalen and Sabellis 1995; Brown et al. 2002; Mideo 2009). However, only few studies investigated multiple infection in the context of parasite that manipulate the phenotype of their host (but see Haine et al. 2005; Vickery and Poulin 2010; Salvaudon et al. 2013). In the *L. boulardi*‐LbFV system, we identified a viral strain presenting a 111‐bp deletion that allowed us to test for the existence of multiple infections. The occurrence of multiple infections observed here and the outcomes in terms of virus transmission may likely affect the fitness reward of the behavioral manipulation from the virus' point of view and therefore influence the evolution of this extended phenotype in nature (Gandon et al. 2006; Varaldi et al. 2009).

The molecular marker used in this study to detect the viral infection has been shown to be highly transcribed in parasitoid females infected by the reference strain LbFV1 and to encode a putative 205 amino acid protein (Patot et al. 2009). As the deletion in LbFV2 does not change the open reading frame of the mRNA, we expect LbFV2 to produce a shorter protein. However, no difference was detected between LbFV1 and LbFV2 regarding their vertical and horizontal transmission rates in single and coinfection or their effect on phenotypic traits including the intensity of the manipulation. Thus, LbFV2 seems not to be defective in any important viral traits and the biological function of the putative viral protein remains unknown. However, the nondeleted sequence of LbFV1 is by far the most prevalent allele found in nature (Patot et al. 2010) and LbFV2 has never been found again in its population of origin (sampled again in 2008, 2009, and 2010), nor in other populations. It is possible that LbFV1 has a selective advantage over LbFV2 but that this advantage could not be detected in our laboratory experiments. Finally, the occurrence of insertion and deletion is a common mutational event in DNA viruses (Coulson and Upton 2011). The presence of the deleted variant in parasitoid populations may thus reflect the recurrent appearance of this deletion that could be favored by the presence of the repeated DNA motif and be maintained through a mutation/drift or a mutation/selection balance.

Our results indicated that an infected parasitoid is still a potential host for a horizontally transmitted viral strain. Indeed, when the recipient parasitoid line was already infected, horizontal transmission often led to coinfection or even to the replacement of the resident strain. However, we cannot ensure that the few cases of replacements that we observed were due to a true mechanism of superinfection, that is, a competitive exclusion of a viral strain through within‐host competition (as defined in Nowak and May 1994). Indeed, apparent replacements could be a consequence of the incomplete vertical transmission of LbFV. Nevertheless, our data showed that one viral strain can replace a resident one (within the parasitoid maternal line) after a few generations of vertical transmission. Interestingly, the efficiency of horizontal transmission was greatly reduced when the recipient parasitoid line was already infected, showing that resident viral strains have a competitive advantage over newly acquired infections. Therefore, LbFV infection confers protection against additional infections, as documented in many host–parasite systems (Hutchison and Sinsheimer 1971; Simon et al. 1990; Nethe et al. 2005; Huang et al. 2008). Such a protection against new infections could be mediated by cross‐immunity mechanisms, which means that parasites induce an immune reaction preventing any additional infection (Raberg et al. 2006), through direct interference among viral strains or through competition for host resources. A consequence of this protective effect could be that in populations showing high prevalence of LbFV, the opportunities for horizontal transmission are limited, which in turn would select for a reduced investment in the manipulation (Varaldi et al. 2009). In southeastern France, strong variation in viral prevalence is observed (Patot et al. 2010), giving the opportunity to test such a hypothesis in the future. However, the protection was not complete, giving some opportunities for the two viral strains to coexist within the same individual host at least for a few generations, and thus allowing them to interact. In terms of virulence, this means that between‐strain competition for limited resources should favor “rapaciousness”, that is favoring viral strains that replicate faster, inevitably causing more damages to the host (Brown et al. 2002). Regarding the behavioral manipulation, however, coinfection enables nonmanipulative viral strains to benefit from the effect of manipulative strains on the expression of superparasitism. Such a conflict should lead to the evolution of cheating if manipulation occurs at a significant cost for the virus and may drive the level of manipulation toward lower values as the frequency of coinfection increases in the population (Brown et al. 2002; Vickery and Poulin 2010). Potential costs that might affect the manipulative viral strains could arise from the expression of genes involved in the manipulative phenotype. Manipulation may also require that the virus infects and replicates in specific tissues that do not directly contribute to virus transmission such as the insect nervous system, potentially leading to a trade‐off with infection of the reproductive apparatus and therefore with transmission.

Over one generation of vertical transmission, LbFV1 and LbFV2 were transmitted with the same efficiency, and with a mean rate consistent with previous data (Varaldi et al. 2006a). However, the vertical transmission rate of both viral strains was slightly altered in coinfected parasitoid lines leading to fewer coinfected offspring than expected, suggesting the existence of competition for vertical transmission. This conclusion is reinforced by studying the segregation of the coinfection through four generations of vertical transmission. Indeed, the proportion of coinfected lines after four generations was much lower than expected under the hypothesis of the absence of competition. On the contrary, coinfecting strains were efficiently jointly transmitted in case of horizontal transmission and the horizontal transmission of one strain was not affected by the presence of the other strain. This suggests that viral strains do not compete for the colonization of developing embryos in case of horizontal transmission, contrary to the vertical transmission. This difference in terms of competition between vertical and horizontal transmission may be the consequence of different mechanisms of transmission. Interestingly, the virus infects the ovaries, where viral replication has been observed, but also the venom gland (Varaldi et al. 2006c) which contains a complex set of proteins that are injected into the host in addition to the egg. One may speculate that the virus population present in the ovaries undergoes a strong bottleneck before being able to reach the developping eggs, but that this bottleneck does not affect the colonization of the venom gland. In both cases, the virus may colonize the developing egg by binding to cellular receptors. However, this colonization would be preceded by a strong bottleneck in the case of vertical transmission. This binding may explain why a previous infection strongly reduces the probability of establishment of another strain through horizontal transmission. On the contrary, an uninfected egg may bear a sufficient number of available receptors to be colonized by several strains by horizontal transmission.

Altogether, our results bring new light on the epidemiology of LbFV. First, coinfection seems to be a common feature in LbFV as it was observed both in nature and in laboratory conditions. In natural populations, the frequency of coinfection may depend on an equilibrium between the acquisition of coinfection through horizontal transmission and the loss through incomplete vertical transmission and within‐host competition. However, the protection conferred by LbFV against additional infections should limit the extent to which coinfection occurs.

Predicting the evolution of the level of manipulation with such complex epidemiological features is not straightforward and needs more theoretical work. The ESS model on the evolution of manipulation (Gandon et al. 2006; Varaldi et al. 2009) should be extended to integrate the existence of coinfection and the resulting social dilemma for the expression of the behavioral manipulation. The great variation of several ecological parameters observed in natural populations of *L. boulardi* will then allow to test the predictions of this model (Fleury et al. 2009; Patot et al. 2010). More generally, the presence of both behavioral manipulation and multiple infections make the *L. boulardi*‐LbFV association an ideal system to test challenging evolutionary hypotheses related to conflict and cooperation in manipulative parasites.

## Conflict of Interest

The authors declare no conflict of interest.

## Supporting information


**Appendix S1.** R script used for the modelization of vertical transmission of the coinfection.
